# An Artificial Intelligence Approach for Gears Diagnostics in AUVs

**DOI:** 10.3390/s16040529

**Published:** 2016-04-12

**Authors:** Graciliano Nicolás Marichal, María Lourdes Del Castillo, Jesús López, Isidro Padrón, Mariano Artés

**Affiliations:** 1 Department of Mechanics, Universidad Nacional de Educación a Distancia (UNED), C/. Juan del Rosal 12, 28040 Madrid, Spain; mlcastillo@ind.uned.es (M.L.D.C.); martes@ind.uned.es (M.A.); 2 Escuela Politécnica Superior de Ingeniería, Universidad de La Laguna, 38001 Tenerife, Spain; ipadron@ull.es; 3 Department of Mechanical Engineering, Universidad Carlos III de Madrid, Madrid 28911, Spain; jelopez@ing.uc3m.es

**Keywords:** Condition monitoring, vibration, Genetic Neuro-Fuzzy systems, fuzzy logic, AUVs

## Abstract

In this paper, an intelligent scheme for detecting incipient defects in spur gears is presented. In fact, the study has been undertaken to determine these defects in a single propeller system of a small-sized unmanned helicopter. It is important to remark that although the study focused on this particular system, the obtained results could be extended to other systems known as AUVs (Autonomous Unmanned Vehicles), where the usage of polymer gears in the vehicle transmission is frequent. Few studies have been carried out on these kinds of gears. In this paper, an experimental platform has been adapted for the study and several samples have been prepared. Moreover, several vibration signals have been measured and their time-frequency characteristics have been taken as inputs to the diagnostic system. In fact, a diagnostic system based on an artificial intelligence strategy has been devised. Furthermore, techniques based on several paradigms of the Artificial Intelligence (Neural Networks, Fuzzy systems and Genetic Algorithms) have been applied altogether in order to design an efficient fault diagnostic system. A hybrid Genetic Neuro-Fuzzy system has been developed, where it is possible, at the final stage of the learning process, to express the fault diagnostic system as a set of fuzzy rules. Several trials have been carried out and satisfactory results have been achieved.

## 1. Introduction

Maintenance is an important aspect of ensuring a satisfactory level of reliability in machinery during its useful life. In these last decades, computer and sensor technologies have experienced rapid development, becoming more powerful and less expensive. In this sense, new maintenance strategies, such as the condition-based maintenance (CBM), have been widely extended [1]. CBM is a maintenance strategy where maintenance actions, based on the collected information by condition monitoring, are recommended [1,2]. These new approaches have aroused great interest. In fact, companies in the naval sector, such as Wärtsilä or SFK, offer new CBM services.

It is known that the CBM programs provide a wide set of advantages. Among them, the reduction of machine maintenance costs and the improvements in on-board safety are typical examples. Furthermore, it minimizes environmental impact through good machinery condition and maximizes vehicle availability. However, one of the key characteristics is to improve equipment reliability and predictability.

An essential point in a CBM maintenance program is the condition monitoring strategy. Although several types of data are used in condition monitoring, vibration data are one of the relevant fault-detection information sources. In fact, some researchers have proposed some strategies based on condition monitoring using vibration data in order to determine faults in different mechanical parts, including bearings, gears, *etc*. [3,4,5,6,7,8,9,10,11,12].

In parallel with the growing interest in CBM, in the last few years, an increasing number of unmanned underwater vehicles and unmanned aerial vehicles have entered the market [13,14,15]. Several commercial manufacturers are interested in this growing market. These vehicles are considered a useful tool because they spread current measurement methods over ground, sea and sky. Some of them are able to travel long distances or dive to low depths in the ocean for many months. A typical scientific mission of an unmanned underwater vehicle could last 90 days, traveling a distance of 2300 km, with immersions of 1000 m depth range [14]. It is important to remark that recovery, because of equipment malfunctioning, relies on ships in this case. This fact increases the operation costs. Hence, it is necessary to assure a high level of reliability and predictability on the on-board equipment. The propulsion system of a great number of AUVs (Autonomous Unmanned Vehicles), in particular, unmanned underwater vehicles and unmanned aerial vehicles are based on propeller-driven propulsion, including a motor, a gear reduction and a propeller. These propulsion systems should operate near maximum efficiency. These are common problems in AUVs, especially in unmanned helicopters, where security and efficiency requirements demand new techniques in order to assure a good behavior. In this sense, many researchers have proposed new fault diagnostic techniques and strategies of fault tolerance control methods for AUVs (underwater and aerial) [16,17,18]. One of the critical components are the gears used in the transmission system in the propeller-driven systems. Efficiency and security depend on the good health of these components. Thus, it is necessary to devise new strategies in order to detect these kinds of fault.

In this paper, a condition monitoring strategy over the gears of a single propeller of an unmanned helicopter will be considered. Moreover, the studies will be focused on the detection of faulty spur gears. In particular, the efforts will be carried out in the case of the determination of defects in small polymer gears, as is the case in small AUVs (underwater and aerial). Note that, although much effort has been made by the scientific community in order to determine defects in gears [4,5,19], little effort has been done in these kinds of gears [20]. Furthermore, an increase usage of polymer gears has been seen in these kinds of vehicles because they are cost-effective, operate without lubrication, contribute little to the total weight of the vehicle, have the ability to operate under conditions of high load and/or high rotation speed and have the ability to avoid material corrosion in the marine environment, which make them appropriate for these applications. However, the stiffness of these polymer gears is lower than that of the metal gears. This fact introduces new challenges in the fault diagnostics since the weakness of the signals obtained by the sensors makes the analysis of the acceleration response spectrum more difficult. Thus, an intelligent condition-monitoring approach will be proposed such that an improvement in the processing data and decision-making phase of the CBM programs can be achieved.

It is important to state that unmanned helicopters, and AUVs in general, are being used everyday in a wider range of military and civilian applications [13]. In particular, the operation advantages of the unmanned helicopters given their characteristics, including vertical takeoff and landing and flexible features of flight, among others, make them an attractive tool in different applications such as search and rescue missions. However, these tasks have to be developed in a secure way. The small size, lightweight and lack of redundancy in sensors and actuators, usual in these kinds of vehicles, can produce a catastrophic accident in the occurrence of a gear fault. Moreover, since the durability of the polymer gears is inferior to metal gears, it is advised to monitor these critical components more frequently. Although one of the most important advantages of these vehicles is that it is not necessary to have a pilot on board, it is possible to cause serious damages to high definition cameras or expensive scientific measurement instruments on board, sometimes several times more expensive than the helicopter. Moreover, damages could be done to people and objects in the operation zone. Hence, new approaches in the field of fault diagnostics are necessary as a tool to ensure functionality and security. In fact, these new approaches are essential in order to improve the efficiency of the fault tolerant control methods [16,17,21,22,23].

In Section 2, the experimental setup along with the acquisition process of the vibration data and the data processing will be depicted. A brief description of the used intelligent approach will be shown in Section 3. The application of the shown methods and the achieved results will be presented in Section 4. Finally, conclusions will be discussed in Section 5.

## 2. Experimental Setup

**Figure 1:**
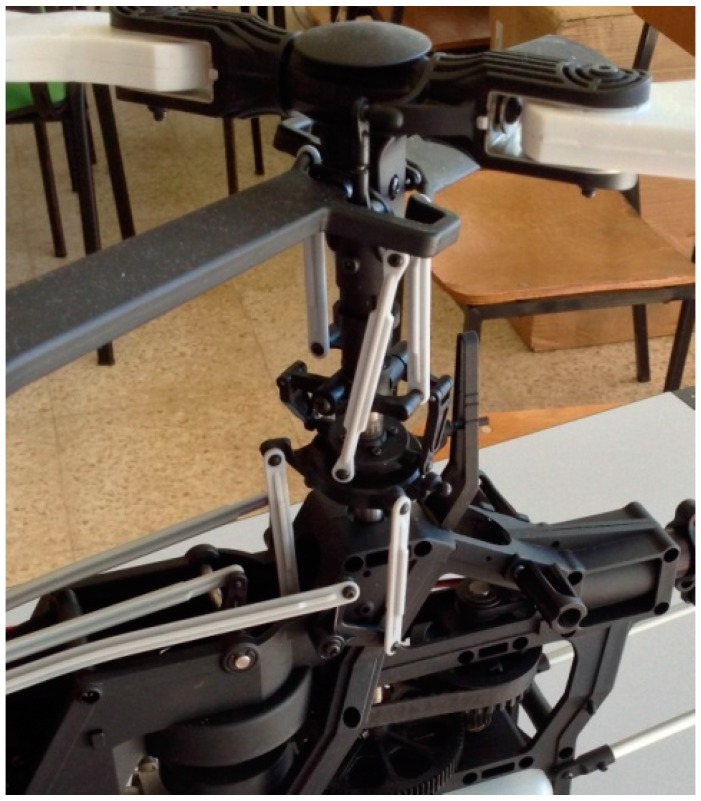
Propeller-driven propulsion system for an unmanned helicopter.

In Figure 1, a typical gear set configuration for a propeller-driven propulsion system in an unmanned helicopter can be seen. Propeller-driven propulsion systems incorporate a gear unit. It is important to remark that the gears are essential components in order to ensure the satisfactory behavior of the whole system. In particular, the experiments will be carried out using the spur polymer gears of the helicopter Align T-Rex 700.

**Figure 2:**
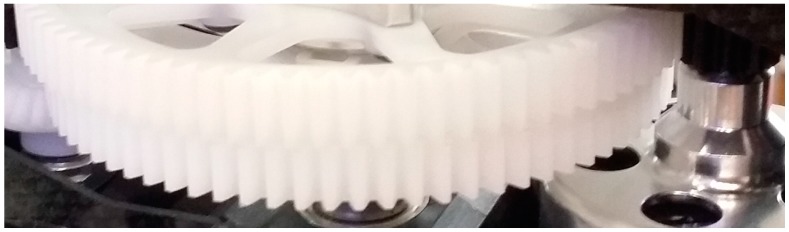
Gear set configuration for the helicopter Align T-Rex 700.

In Figure 2, the main drive gear HN7019t-1 and the Engine Gear Set HN7037T for this helicopter’s propeller-driven propulsion system can be seen. Note that these spur gears have been considered in this work, given these helicopters have an open design. That is, most of the components are easy to access, in particular the gears, as shown in Figure 2. However, in the case of unmanned underwater vehicles, the structure is closed, given being watertight is a very important feature in these kinds of vehicles. Moreover, the availability of obtaining a wide stock of spare parts at reasonable prices allows tackling the trials in an easier way.

In order to detect incipient defects in these components the vibrations of the system could be measured by placing an accelerometer near the whole system. In this sense, a prototype has been devised in order to study the vibration-signal characteristics and their relations with the different possible defects. Over this gear test bench, necessary modifications to suit transmissions considered and other types of transmissions (especially transmissions with internal gears) have been made. In fact, others adaptations have been prepared, permitting the performance of dynamic analysis of load distribution along the tooth.

This testing equipment allows using different spur gears and to analyze their vibrational response. In fact, health spur gears and faulty spur gears have been tested. The vibration signal is collected by a piezoelectric accelerometer, in this case, the accelerometer of Brüel & Kjaer 4383. Moreover, the accelerometer signal is amplified by the Nexus system of Brüel & Kjaer and is registered by the Multi-analyzer System Type 3560. Finally, it is sent to a laptop where the data are saved and the signal-processing phase is carried out. The data have been registered with a sampling time of 30.51 ns. In addition, to collect the accelerometer data, it is necessary to process these data; that is, a signal-processing step is necessary. In particular, a time-frequency method has been chosen in this work. In fact, the short-time Fourier transform has been used. Note that time-frequency domain methods such as the short-time Fourier Transform provide more information and are convenient in this case because a non-stationary signal is considered as a series of adjacent quasi-stationary signals. Furthermore, it is desired that the devised diagnostic approach can determine the kind of defect, in addition to detect if a gear is faulty.

**Figure 3:**
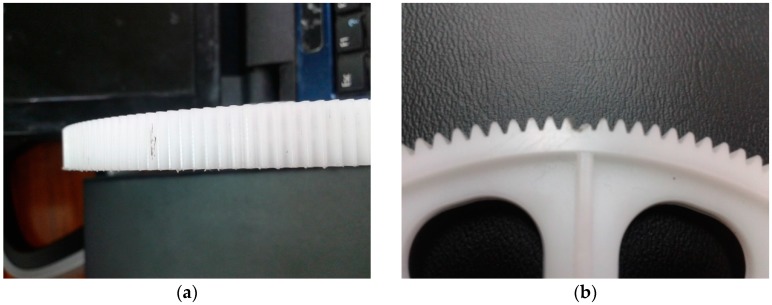
(**a**) Faulty gear with scuffing; and (**b**) faulty gear with broken tooth.

Thirty gears have been chosen to carry out the study. Ten gears have been chosen without defects and the others have some kind of defect. For the sake of simplicity, only the following set of faulty gears have been considered: ten with scuffing defects and the rest of them with a broken tooth. Photographs of both kinds of gear faults can be seen in Figure 3.

**Figure 4:**
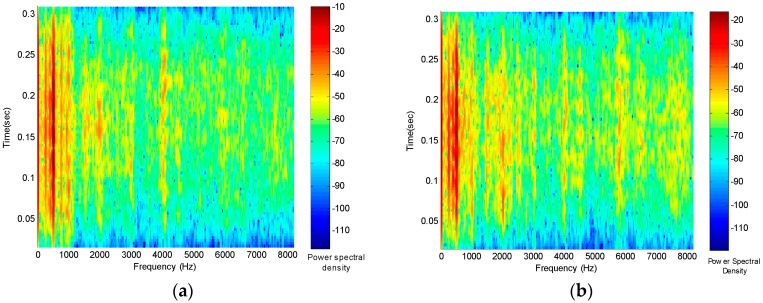
(**a**) Short-time Fourier Transform for a gear with a partially damaged tooth; and (**b**) short-time Fourier Transform for a healthy gear.

Different captured vibration signals have been processed by Short-Time Fourier Transform. In Figure 4a, the result for a faulty gear, where the variation of the power spectral density in the time-frequency domain can be seen. Furthermore, the result corresponding to a healthy gear is shown in Figure 4b.

The next step in devising a diagnostic approach would be to analyze these graphs in order to look for relevant characteristics that could be directly related to the specific defect in the gear, or at least to distinguish between normal and faulty gears. As can be seen from Figure 4a,b, this task is not easy. It is difficult to find common characteristics for faulty or healthy gears, even though a signal-processing step has been applied and the study has been restricted to two kinds of defects in this case. Moreover, an automatic diagnostic approach would be desirable. In this sense, an intelligent diagnostic approach will be presented in this paper in order to advance towards this target. In Section 3, a brief description of the techniques used to improve the decision mechanism will be presented.

## 3. Artificial Intelligence Approach

In recent years, different approaches based on artificial intelligence techniques have been proposed. In the case of fault diagnostics, the works of different researchers can be mentioned [4,24,25,26,27,28,29]. Several techniques have been applied inside the artificial Intelligence field. Among them, the paradigms of the Neural Networks, the Fuzzy Logic, the Expert Systems and the Genetic Algorithms have been widely used [24,25,28,29]. In this paper, effort will be focused on the Neuro-Fuzzy systems [30,31]. This paradigm could be considered a hybrid approach between Neural Networks and the Fuzzy Systems. It is important to remark that only a Neuro-Fuzzy system approach will be considered in this paper, even though other combinations of two paradigms are possible, such as the paradigm known as Fuzzy Neural Networks according to Mitra *et al.* [32]. Note that the Neuro-Fuzzy system approach allows combining the two essential advantages of both paradigms. That is, the resultant systems include the inherent properties of learning corresponding to the Neural Networks and the symbolic properties of the Fuzzy Logic systems, given that the final system can be expressed by rules. This is an appreciated feature when dealing with fault diagnostic systems. Thus, a Neuro-Fuzzy system has been chosen instead of a Fuzzy Neural Network because, as pointed out by Mitra *et al*. [32], this first kind of hybridization between Neural Networks and Fuzzy systems allows expressing the final system according to the fuzzy logic formalism. In the following, the structure of the Neuro-Fuzzy system and the learning mechanism will be presented.

### 3.1. Structure of the Neuro-Fuzzy System

**Figure 5:**
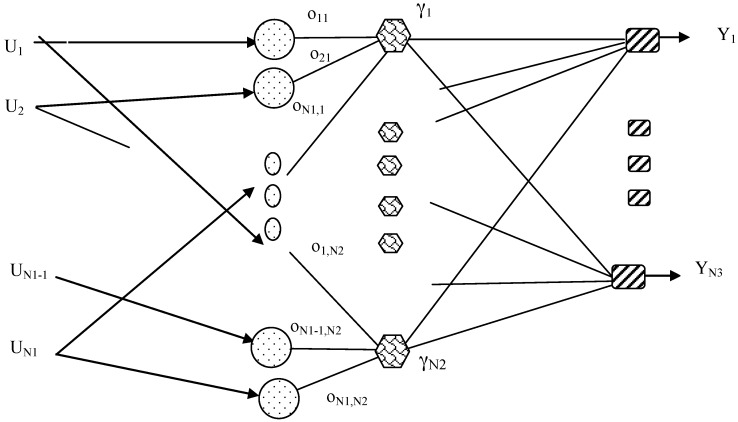
Structure of the Neuro-Fuzzy system.

The structure used is a Neuro-Fuzzy system based on the system proposed by Jang [33], known as the Adaptive Neuro-Fuzzy Inference System. In this sense, several similar structures have been proposed [32,33,34,35,36]. In particular, a structure of a Neural Network consisting of three layers will be used in this work. In Figure 5 a diagram of this structure is shown [33]. As can be seen, the system has *N*1 inputs and *N*3 outputs. 

The equations of the Neuro-Fuzzy system nodes are:

(1) $$o_{ij}=\exp\left(-\frac{{\left({\;U}_{i}-m_{ij}\right)}^{\;2}}{{\sigma_{ij}}^{2}}\right)\;\begin{array}{c} j=1,2,\;\ldots ,N2\\ i=1,2,\ldots ,N1 \end{array}$$

(2) $$\gamma_{j}=\;\min\left[o_{1j},o_{2j},\;\ldots \;,o_{ij},\;\ldots \;,\;o_{N1j}\right]\;j=1,2,\ldots ,N2$$

(3) $$Y_{k}=\frac{\sum_{j}{sv}_{jk\;}\gamma_{j}}{\sum_{j}\gamma_{j}}\;\begin{array}{c} j\;=1,2,\;\ldots ,\;N2\\ k=1,2,\ldots ,N3 \end{array}$$

where ${\;U}_{i}$ = *i-*th Input to the Neuro-Fuzzy System; $Y_{k}$ = *k*-th output of the Neuro-Fuzzy System.

As can be seen, the structure of the Neuro-Fuzzy system can be considered a mathematical model where the output values are obtained according with the values of the inputs. Furthermore, the output values also depend on several of the Neuro-Fuzzy system parameters. In this case, they depend on the parameters $m_{ij}$, $\sigma_{ij}$, ${sv}_{jk\;}$ and *N*2.

Thus, it is important to devise a learning algorithm that is able to determine this set of parameters in order to achieve a mathematical model that can solve the fault diagnostic problem in the best way. Therefore, no assumptions over a predefined dynamic model have been set. In the following section, the learning algorithm used in this work will be depicted in detail.

### 3.2. Proposed Learning Algorithm

The learning algorithm has been divided into three steps. The first phase is focused on the application of an unsupervised learning algorithm to obtain initial values for the parameters $m_{ij}$, and ${sv}_{jk\;}$, while, the parameters *N*2 and $\sigma_{ij}$ are determined by a genetic algorithm in the second phase. Finally, $m_{ij}$, $\sigma_{ij}$, and ${sv}_{jk\;}$ are adjusted by a supervised learning algorithm in the third phase. It is important to remark that the proposed system can be considered a Genetic-Neuro-Fuzzy system, given a genetic algorithm is applied in the adjusting phase of the parameters. In the following, it will be named the Genetic Neuro-Fuzzy system. It is important to remark that the Genetic phase is an essential step in the proposed approach given it determines the definition of the number of hidden layer nodes in the final structure of the system.

#### 3.2.1. Unsupervised Learning Phase

The first phase is focused on the determination of the initial values for the parameters $m_{ij}$ and ${sv}_{jk\;}$. In order to set these parameters, a Kohonen’s self-organizing feature map algorithm is applied [37,38]. The vectors of the nodes corresponding to the self-organizing map are chosen as follows:

(4) Wj = (w1j, w2j, w3j, …, w(N1+N3) j)    j = 1,2,…,N2

Note that, a dimension of *N*1 + *N*3 has been chosen for these nodes. Furthermore, the inputs to the self-organizing map are expressed as follows:

(5) V = (U1, U2, …, UN1,Y1, Y2, …, YN3)

where (*U*1, *U*2, …, *UN*1) = Input vector to the Neuro-Fuzzy System, (*Y*1, *Y*2, …, *YN*3) = Desired output vector.

As can be seen above, a 1-D Kohonen self-organizing map is used; that is, only a layer of nodes has been considered. In addition, the Kohonen auto-organizative algorithm [37] has been applied to update the weights in Equation (4). As is known, this learning algorithm is a typical unsupervised learning algorithm. After the application of the unsupervised learning algorithm has been concluded, an assignment of the parameters $m_{ij}$, and ${sv}_{jk\;}$ is carried out as follows:

(6) $$m_{ij}=w_{ij}\;\begin{array}{c} j=1,2,\;\ldots \;,\;N2\\ i=\text{1,2} \end{array}$$

while the values for the estimated outputs are chosen using the rest of components of the same vector as

(7) $${sv}_{jk}=w_{N1+k\;,\;j}\;\begin{array}{c} \;k=1,\text{​}2\\ j=\text{1,2},\;\ldots \;,\;N2 \end{array}$$

Note that this assignment is only an initial assignment of values for these parameters; that is, these parameters are updated in new phases of the learning algorithm. However, this initial assignment is an important step in order to achieve better results for the algorithm. The next phase will be focus on the determination of the number of rules and the values of ${sv}_{jk\;}$.

#### 3.2.2. Genetic Algorithm Phase

The values for the parameters $\sigma_{ij}$ are necessary to build the Genetic Neuro-Fuzzy system. Moreover, it would be convenient to design a method to reduce the number of nodes at the hidden layer, *N*2. It is important to remark that *N*2 could also be seen as the number of fuzzy rules. If it is considered the structure of the Genetic Neuro-Fuzzy system shown in Section 3.1, it could be seen that a set of nodes at the input layer, together with a set of nodes at the output layer and one node at the hidden layer, build a fuzzy rule, after the parameters of the Genetic Neuro-Fuzzy system are fixed. That is, each node at the hidden layer determines a different fuzzy rule; hence, the number of these nodes determines the whole number of rules.

Taking into account the considerations done in the previous paragraph, this learning phase will be focused on two targets. The first one is to determine adequate values for the parameter ${sv}_{jk\;}$ and second one is to achieve a reduced number of nodes at the hidden layer. Note that this last target consists of selecting the most adequate fuzzy rules in order to reduce the number of nodes; that is, the reduction of nodes is carried out with a rational criterion based on the associated rules of the nodes.

As is known, the Genetic Algorithm [39,40] is an algorithm based on the biological paradigm of genetic evolution. Thus, it is essential to define the information corresponding to an individual from basic information, known as gene. A vector will be considered in this paper that represents the individual, and the vector components are the genes. In this context, the components of each vector (individual) consist of a representation of the different hidden nodes by a Boolean value and the $\sigma_{ij}$ values. That is, each individual is a vector as follows:

(8) $$V=\left(\begin{array}{l} 0\;or\;1,0\;or\;1,0\;or\;1,\ldots ,\;\sigma_{1,1},\;\sigma_{1,2,},\;\sigma_{1,3,},\ldots ,\;\sigma_{1,N2},\;\\ \sigma_{2,1,},\sigma_{2,2,},\ldots ,\;\sigma_{2,N2},\ldots ,\sigma_{N1,1,}\sigma_{N1,2,},\ldots ,\sigma_{N1,N2,} \end{array}\right)$$

As shown in Equation (8), the first N_2_ components of the vector *V* are binary values. A one value means this rule is considered in the whole Genetic Neuro-Fuzzy System, whereas a zero value means this rule is eliminated from the whole system and the last *N*1 × *N*2 values correspond to the values $\sigma_{ij}$. The values $\sigma_{ij}$ associated to hidden nodes with zero values will not be considered in the implementation of the final solution, however, they have been included in Equation (8) for the sake of simplicity. In this way, several individuals are created. A fitness function is defined taking into account the evaluation of the error between the real output values and the individual output values. Moreover, each individual is a possible trained Genetic Neuro-Fuzzy system and the values obtained in Equations (6) and (7) are taken for all individuals in this learning phase. As a result of the application of the genetic algorithm over these individuals satisfactory values are obtained for $\sigma_{ij}$ values and an adequate set of *N*2 rules (nodes at the hidden layer) are also obtained. Several iterations are carried out by the genetic algorithm in order to obtain the best individual. In particular, a genetic algorithm with a population of 50 individuals has been chosen and iterations until 100 generations have been considered. In addition, operators of crossover and mutation have been applied in each generation, considering a 0.2 probability of crossover-operator application *versus* a 0.8 probability of mutation-operator application in order to look for a greater variability in the parameters. Although with the application of this phase a complete set of Genetic Neuro-Fuzzy system parameters is achieved, new adjustment learning algorithms could be applied in order to refine these values, obtaining a better final Genetic Neuro-Fuzzy system. 

#### 3.2.3. Supervised Learning Phase

This last phase is focused on improving the selection of the parameters $m_{ij}$, $\sigma_{ij}$ and ${sv}_{jk\;}$ of the Genetic Neuro-Fuzzy system. If it is considered that the Genetic Neuro-Fuzzy system could be seen as a Neural Network build on three layers, the standard learning algorithms adapted to the mathematical expressions of these particular nodes could be applied. The nodes at the input layer have the same mathematical expression as the neurons in a Radial Basis Neural Network; hence, it is possible to use the least mean squared learning algorithm as usual in a typical Radial Basis Network [41]. In order to obtain the expression in this case, it is necessary to consider the error function between the outputs of the Genetic Neuro-Fuzzy system and the real outputs of the available patterns. This expression is as follows:

(9) $$E=\;\frac{1}{2}\;\sum_{k=1}^{N3}\left(Y_{k}-S_{k}\right)\;k=1,\;\ldots ,\;N_{3}$$

where *S*_k_ = *k*-th output of the Genetic Neuro-Fuzzy System, *Y*_k_ = *k*-th real output.

The target of the algorithm is to choose the parameters of the Genetic Neuro-Fuzzy System such that it is possible to get a low value for the error function. Initial parameters of the Genetic Neuro-Fuzzy system are $m_{ij}$, $\sigma_{ij}$ ${sv}_{jk\;}$ and *N*2, fixed in the previous phases of the learning algorithm. This phase only change these values in order to minimize the error function in order to readjust the previous parameters. The following expression will be applied in an iterative way, according to the least mean squared learning algorithm for each parameter.

(10) $$par(t+1)=par(t)-\text{​}lr\text{​}\frac{\partial \;E}{\partial \;par}\;t=1,2,\ldots ,\;f$$

where *par* = it is the parameter to adjust ($m_{ij}$, $\sigma_{ij}$ or ${sv}_{jk\;}$), *t* = the number of iteration, *f* = the maximum number of iterations, *lr* = learning rate (constant value).

A maximum number of iterations of about 100,000 has been considered in most cases, although in some cases a smaller number of iterations has been chosen, taking into account the small magnitude of the error-function values.

Once this learning phase has been applied, a final Genetic Neuro-Fuzzy system is achieved. The resultant Genetic Neuro-Fuzzy system could be considered the standard Fuzzy Logic System, where a set of fuzzy rules has been defined.

## 4. Application of the Artificial Intelligence Approach

The techniques depicted in Section 3 have been applied to detect defects in gears. In order to test the algorithm, several samples have been prepared and the vibration spectra produced by these normal and faulty gears have been collected and processed. However, it is necessary to apply a final algorithm in order to decide from these data if a gear is normal or faulty. Moreover, two Genetic Neuro-Fuzzy systems have been used, where first one is focused on deciding between normal and faulty gear and the second one is focused on deciding in a faulty gear if the defect is a scuffing defect or a broken tooth defect. It is important to remark that this structure of the whole system allows simplifying the learning process, dividing into two Genetic Neuro-Fuzzy systems the process of taking a classification decision.

The first step to apply the Genetic Neuro-Fuzzy system is to adapt the time-frequency domain response to the inputs of the system. As can be seen in Figure 4, the time-frequency domain responses are represented as a two-dimensional graph. Therefore, the response is a function depending on the time and frequency, given the short-time Fourier transform has been applied over the captured data by the accelerometer. Thus, 2D graphs are achieved, as shown in Figure 4. However, it is necessary to obtain a set of representative values to be used as the inputs of the Genetic Neuro-Fuzzy System.

In this paper, the 2D graphs have been divided into a set of squared areas, where the maximum function value of each square has been chosen as the inputs to the Genetic Neuro-Fuzzy System. Maximum values have been chosen given the resonance response of the gears is a significant feature of the mechanical system. Therefore, it is necessary to divide the time and frequency interval into equal parts in order to obtain these squared areas. These equal parts will be named bands in the following. In the case of this work, intervals of 15 components for the time and frequency have been chosen to build a band. Hence, a total number of 108 bands are built, corresponding to 108 inputs of the Genetic Neuro-Fuzzy system. This is an adequate number of inputs, given a higher number of inputs makes the learning process more complex, whereas, if a smaller number of inputs were chosen, essential information for the classification process could be lost.

Several trials have been carried out. In Table 1, the outputs corresponding to the samples used for the training process, once the final Genetic Neuro-Fuzzy is achieved, are shown. The Genetic Neuro-Fuzzy system has two outputs. If the first output is near 1, the corresponding gear is classified as normal, whereas if it is nearer to 0, it is classified as faulty. Furthermore, the values of the second Genetic Neuro-Fuzzy output are the complementary values, that is, the result of the operation of one minus the first output of the Genetic Neuro-Fuzzy system. Thus, if the first output is greater than the second output, the corresponding gear is normal. On the contrary, if the second output is greater than the first output, the corresponding gear is faulty. The differences between these two outputs have been observed without fixing a threshold in order to accomplish the classification process. It is important to remark that if these differences are greater it could be considered that the classification is accomplished in a more appropriate way. As can be seen in Table 1, all training patterns are classified correctly. However, it is observed that the differences between the two outputs are different for each pattern. This variable could be taken as an indicator of the difficulty level for classifying a particular pattern. It is the case of Pattern 10, where only a difference less than 0.1 is achieved. Although the classification has been done correctly, it is observed that there is a high degree of difficulty by the Genetic Neuro-Fuzzy System for classifying this particular pattern. In this sense, it would be convenient if the final fault decision system provides these difference values as an indicator of reliability in each particular classification.

In Table 2, the results corresponding to testing patterns are shown. In this case, only one classification error is achieved (it is pointed out using bold letter). Note that eighty percent of the samples have been used in the training set, while the rest have been used to verify the generalization properties of the final system. In this case, 300 rule nodes have been considered, while 100,000 iterations have been carried out in the supervised learning algorithm. Furthermore, eighty percent of the sample set have been considered in the learning process, while the rest of the samples have been considered for testing the generalization properties of the final system. In this case, a squared mean error of 0.019003 has been achieved for the training patterns and a squared mean error of 0.11372 has been achieved for the testing patterns.

On the other hand, a Genetic Neuro-Fuzzy system has been trained in order to distinguish between a gear with a scuffing damage and with a broken tooth. This case also used a similar proportion between the samples used in the learning process and the samples used to verify the generalization properties of the Genetic Neuro-Fuzzy system; that is, eighty percent *versus* twenty percent. As can be seen in Table 3, all training samples are classified correctly in this case, after a learning process has been applied to the Genetic Neuro-Fuzzy system. In fact, a squared mean error of 0.015266 has been achieved for the training patterns. Moreover, a squared mean error of 0.03354 has been achieved for the testing patterns. The classification of these samples is shown in Table 4. Note that the training and testing samples are classified correctly in both cases.

## 5. Conclusions

In this paper, the diagnostic of spur gears has been performed. The studies focused on the field of unmanned helicopters, although the results could be extended to unmanned underwater vehicles and other kinds of unmanned aerial vehicles. New applications have been incorporated into these vehicles, where good maintenance is essential to increase their robustness, in hostile environments, such as the sea, where it is expensive and sometimes not possible to perform maintenance operations, or in search and rescue missions where ensuring efficient and secure conditions is an essential characteristic. In particular, a set of spur polymer gears corresponding to a single propeller system have been taken for the experiments and two kinds of defects have been introduced: scuffing defects and broken tooth defects. Furthermore, testing equipment has been adapted to carry out the experiments. The testing equipment has been adapted to use the main drive gear and the engine gear set of the propeller-driven system of the helicopter Align T-Rex 700. In this case, small-size polymer gears have been considered *versus* most studies in this field that have mainly been devoted to gearboxes with metal gears. However, polymer gears introduce new challenges to fault diagnostics since the obtained signals by the sensors are more difficult to analyze. A system based on an intelligent approach to detect the incipient faulty gears has been presented in this paper. Moreover, the shown methods are able to determine the specific kind of defect. These alternative techniques are based on learning properties that allow capturing the complex features of the patterns to classify, as it is difficult to obtain the common characteristics of these patterns using other techniques. In this paper, an alternative proposal combining different artificial intelligence paradigms has been presented for use as a fault diagnostic system. In fact, a hybrid Genetic Neuro-Fuzzy system has been devised where it is possible in the final stage of the learning process to express the fault diagnostic system as a set of fuzzy rules. Note that the used structure and learning algorithms allow keeping the final system in fuzzy-logic formalism. This is an interesting feature for a fault decision system, where the design of an automatic system is desirable. Additionally, the proposed Genetic Neuro-Fuzzy system approach introduces mechanisms to reduce the number of rules in their learning algorithms and its structure allows operating with a large number of inputs. This last feature is much appreciated in classification problems, where a large number of inputs to the Genetic Neuro-Fuzzy system is generated. Several trials have been carried out with the shown system and satisfactory results have been achieved.

## Figures and Tables

**Table 1:** Output values of the Genetic Neuro-Fuzzy system for the training set.

Vibration Patterns Used as a Training Set	Real Type	First Genetic Neuro-Fuzzy Output (Healthy or Normal)	Second Genetic Neuro-Fuzzy Output (Faulty)
1	Normal	0.99965	0.00035222
2	Normal	1	1.2122 × 10^−11^
3	Normal	0.9963	0.0036951
4	Normal	0.9181	0.081903
5	Normal	0.91383	0.08617
6	Normal	0.99791	0.0020946
7	Normal	1	5.0458 × 10^−8^
8	Normal	0.71725	0.28275
9	Faulty	1.6529 × 10^−5^	0.99998
10	Faulty	0.46885	0.53115
11	Faulty	1.7053 × 10^−32^	1
12	Faulty	5.7391 × 10^−18^	1
13	Faulty	2.407 × 10^−11^	1
14	Faulty	0.038761	0.96124
15	Faulty	3.6484 × 10^−12^	1
16	Faulty	0.0046506	0.99535
17	Faulty	4.982 × 10^−9^	1
18	Faulty	2.1034 × 10^−24^	1
19	Faulty	5.0964 × 10^−24^	1
20	Faulty	0.3148	0.6852
21	Faulty	0.20375	0.79625
22	Faulty	1.177 × 10^−6^	1
23	Faulty	4.6292 × 10^−18^	1
24	Faulty	0.0011171	0.99888

**Table 2:** Output values of the Genetic Neuro-Fuzzy system for the testing set.

Vibration Patterns Used as a Training Set	Real Type	First Genetic Neuro-Fuzzy Output (Healthy or Normal)	Second Genetic Neuro-Fuzzy Output (Faulty)
1	Normal	0.65369	0.34631
2	Normal	0.99947	0.00052813
3	Faulty	0.74994	0.25006
4	Faulty	3.3941 × 10^−94^	1
5	Faulty	2.2563 × 10^−42^	1
6	Faulty	5.8757 × 10^−12^	1

**Table 3:** Output values of the Genetic Neuro-Fuzzy system for the training set.

Vibration Patterns Used as a Training Set	Real Type	First Genetic Neuro-Fuzzy Output (Scuffing)	Second Genetic Neuro-Fuzzy Output (Broken Tooth)
1	Scuffing	0.93596	0.064036
2	Scuffing	0.99737	0.0026326
3	Scuffing	0.77922	0.22078
4	Scuffing	0.85942	0.14058
5	Scuffing	0.99994	6.2056 × 10^−5^
6	Scuffing	0.92721	0.072786
7	Scuffing	0.96352	0.036479
8	Scuffing	0.99924	0.00075772
9	Broken tooth	6.2106 × 10^−5^	0.99994
10	Broken tooth	0.038157	0.96184
11	Broken tooth	0.0019513	0.99805
12	Broken tooth	0.38519	0.61481
13	Broken tooth	5.661 × 10^−5^	0.99994
14	Broken tooth	0.099974	0.90003
15	Broken tooth	0.0012966	0.9987
16	Broken tooth	0.071967	0.92803

**Table 4:** Output values of the Genetic Neuro-Fuzzy system for the testing set.

Vibration Patterns Used as a Training Set	Real Type	First Genetic Neuro-Fuzzy Output (Scuffing)	Second Genetic Neuro-Fuzzy Output (Broken Tooth)
1	Scuffing	0.63948	0.36052
2	Scuffing	1	6.9317 × 10^−22^
3	Broken tooth	0.064666	0.93533
4	Broken tooth	0.00032829	0.99967

## Abbreviations

The following abbreviations are used in this manuscript:

CBM	condition-based maintenance
AUVs	Autonomous Unmanned Vehicles
